# Using the health belief model to identify barriers to seasonal influenza vaccination among Australian adults in 2019

**DOI:** 10.1111/irv.12843

**Published:** 2021-02-15

**Authors:** Mallory J. Trent, Daniel A. Salmon, C. Raina MacIntyre

**Affiliations:** ^1^ Biosecurity Program The Kirby Institute University of New South Wales Sydney NSW Australia; ^2^ Departments of International Health and Health, Behavior and Society Institute for Vaccine Safety Bloomberg School of Public Health Johns Hopkins University Baltimore MD USA

**Keywords:** Australia, barriers, health belief model, influenza, predictors of vaccination, vaccine hesitancy

## Abstract

**Background:**

Each year tens of thousands of Australians become ill with influenza, resulting in thousands of severe infections that require hospitalisation. However, only 40% of adults receive the annual influenza vaccine. We surveyed Australian adults to provide up to date, population‐specific data on the predictors and barriers of seasonal influenza vaccination.

**Methods:**

We administered an online survey to a nationally representative sample of Australian adults. We designed survey questions using the theoretical constructs of the health belief model. Using simple and multivariable Poisson regression, we identified attitudes and beliefs associated with influenza vaccination in 2019.

**Results:**

Among 1,444 respondents, 51.7% self‐reported influenza vaccination in 2019. We estimated vaccine coverage to be 44% for adults under 45, 46% for adults aged 45 to 64 and 77% for adults aged 65 and over. The strongest individual predictors of self‐reported vaccination were believing the vaccine is effective at preventing influenza (APR = 3.71; 95% CI = 2.87‐4.80), followed by recalling their doctor recommending the vaccine (APR = 2.70; 95% CI = 2.31‐3.16). Common perceived barriers that predicted self‐reported vaccination included believing the vaccine could give you influenza (APR = 0.59; 95% CI = 0.52‐0.67), believing the vaccine can make you ill afterwards (APR = 0.68; 95% CI = 0.62‐0.74) and preferring to develop immunity “naturally” (APR = 0.38; 95% CI = 0.32‐0.45).

**Conclusion:**

Although vaccine uptake in 2019 appears to be higher than previous years, there are perceived barriers which may limit uptake among Australians. Tailored interventions are needed to combat widespread influenza vaccine hesitancy, particularly among high‐risk groups.

## 1 INTRODUCTION

Each year influenza is associated with more than 500 000 deaths globally.^1^ Although annual vaccination for influenza is an effective way to prevent infection and severe disease,^2^ there is widespread hesitancy towards influenza vaccines.^3^, ^4^, ^5^, ^6^, ^7^ In the past two decades, the use of the internet has skyrocketed and it plays an increasingly large and complex role in health decision‐making.^8^, ^9^ This has allowed anti‐vaccine sentiment to enter the mainstream, and as a result, misinformation about vaccines, including the influenza vaccine, is common.^8^

There are a number of psychological barriers that are known to prevent uptake of the influenza vaccine.^3^ These include low perceived risk of disease,^3^, ^10^ low perceived effectiveness of the vaccine,^3^, ^11^, ^12^ belief that the vaccine can make you ill,^3^, ^11^, ^13^ and low perceived behavioural control,^3^ and distrust of the vaccine or health authorities.^3^, ^14^ There are also common contextual barriers to influenza vaccine uptake, including cost,^3^, ^14^, ^15^ access to healthcare services,^3^ frequency of interaction with healthcare services ^3^, ^16^, ^17^ and lack of recommendations from healthcare professionals or other trusted sources.^3^, ^12^, ^13^

In Australia, influenza vaccine coverage among adults remains low compared with routine childhood vaccines.^6^, ^18^ The most recent nation‐wide survey of influenza vaccination among adults, conducted in 2014, found that approximately 40% of adults were vaccinated for influenza that year.^6^ Coverage was 73% among those over age 65 but less than 30% among those under age 50.^6^ Coverage among adults over age 65 increased significantly after the introduction of universal funding for influenza vaccines for this age group in 1999,^11^ but remained relatively constant until 2014, while coverage among younger adults appears to have increased somewhat during the same time period.^6^, ^19^, ^20^

There is limited research on the predictors and barriers of influenza vaccination among Australian adults.^11^ Furthermore, most of this research was conducted many years ago or in specific, high‐risk populations.^11^ Thus, most of the information available on predictors of influenza vaccination among Australian adults may be outdated or not applicable to the general population.

Designing effective, tailored interventions requires a sound understanding of the key drivers and barriers to vaccination. Thus, up to date information on the drivers and barriers of adult vaccination is needed. The purpose of the present study was to provide up to date, population‐specific data on the predictors and barriers of seasonal influenza vaccination among Australian adults.

## 2 METHODS

### 2.1 Study design and recruitment

We conducted an online, anonymous survey in October 2019 of adults aged 18 and over that reside in Australia. Lucid (https://luc.id/), a consumer panel marketplace, distributed the survey to a random sample of adults from probability‐based research panels using their online marketplace. Lucid's research panels are reviewed regularly by third‐party data specialists to monitor data quality and minimise bias. The sample was stratified to be representative of Australia in terms of age, gender, and state or territory of residence. All recruited panel members were screened for inclusion based on age and country of residence and provided informed consent prior to completing the survey. The University of New South Wales Human Research Ethics Committee reviewed and approved this study (HC #190617).

### 2.2 Survey questions

We designed our survey questions using the theoretical constructs of the health belief model (HBM). The HBM explains and predicts health behaviours in terms of an individual's perceived susceptibility to disease, perceived severity of disease, perceived benefits of an intervention, perceived barriers to an intervention, cues to action and self‐efficacy ^21^ and has been used in other contexts to understand vaccination behaviours.^22^, ^23^ We included 16 survey items that related to five theoretical constructs of the HBM. To assess an individual's perceived susceptibility to influenza, we asked “On a scale from 0 to 10, how likely do you think you are to get the flu next year?”. To assess an individual's perceived severity of influenza, we asked “On a scale from 0 to 10, how severe do you think the flu would be if you got it?”. The remaining items were assessed using either 2‐point or 4‐point Likert scales. These items and their associated response options are listed in Table 1.

**Table 1:** Health belief model (HBM) constructs used in survey design

HBM construct	Survey item	Response options
Perceived susceptibility	On a scale from 0 to 10, how likely do you think you are to get the flu next year?	Sliding scale from 0 to 10
Perceived Severity	On a scale from 0 to 10, how severe do you think the flu would be if you got it?	Sliding scale from 0 to 10
Perceived benefits	The flu jab is effective at preventing the flu and keeping me well	4‐point Likert scale (strongly disagree, disagree, agree, strongly agree)
	If I get a flu jab, it will protect my loved ones from the flu	4‐point Likert scale (strongly disagree, disagree, agree, strongly agree)
Perceived barriers	The flu jab can give you the flu	2‐point Likert scale (disagree, agree)
	The flu jab can make you feel sick afterwards	2‐point Likert scale (disagree, agree)
	I don't trust vaccines	4‐point Likert scale (strongly disagree, disagree, agree, strongly agree)
	Myself or someone I know has had a bad experience with a vaccine	2‐point Likert scale (disagree, agree)
	I prefer to develop immunity naturally	4‐point Likert scale (strongly disagree, disagree, agree, strongly agree)
	I am afraid of needles	4‐point Likert scale (strongly disagree, disagree, agree, strongly agree)
	I have difficulties getting an appointment with my doctor to get vaccinated	2‐point Likert scale (disagree, agree)
	Getting the flu jab is expensive	4‐point Likert scale (strongly disagree, disagree, agree, strongly agree)
	I do not have time to get vaccinated	2‐point Likert scale (disagree, agree)
	I have mobility issues that make it difficult to get to my GP	2‐point Likert scale (disagree, agree)
Cues to action	My doctor recommended that I get the flu jab	2‐point Likert scale (disagree, agree)
	I have seen advertisements for the flu jab in pharmacies/chemists	2‐point Likert scale (disagree, agree)

Our primary outcome, self‐reported influenza vaccination in 2019, was assessed using the question, “did you get the flu jab in 2019?” with the following response options: yes, no, not sure. We considered participants as not vaccinated if they answered “no” or “not sure.” We also collected participant data on health status, healthcare utilisation and sociodemographic factors. Our full list of survey questions can be found in the Supporting Information.

### 2.3 Data analysis

We calculated descriptive statistics for all variables of interest. We used Poisson regression with robust error estimation to determine which factors were associated with self‐reported influenza vaccination in 2019. In cross‐sectional studies with common outcome variables, Poisson regression estimates prevalence ratios directly and is believed to outperform logistic regression.^24^ First, we conducted Poisson regression to identify the sociodemographic and clinical factors associated with self‐reported vaccination, both one‐way and adjusted for significant predictors (*P* < .05). Second, we identified the HBM items associated with self‐reported vaccination, using one‐way Poisson regression as well as adjusting for significant (*P* < .05) sociodemographic and clinical predictors.

Third, we conducted multivariable Poisson regression with all five HBM constructs. For constructs with more than one survey item, we tested survey items for internal consistency using Cronbach's alpha.^25^ Constructs with an *α* of 0.6 or greater were analysed as a single scale in regression models. As a result, the two survey items for perceived benefits were combined into a single scale (*α* = 0.68), six items under perceived barriers were combined into a single scale of “psychological barriers” (*α* = 0.65), and four items under perceived barriers were combined into a single scale of “physical/structural barriers” (*α* = 0.64). The two items under cues to action were left as single items (*α* = 0.43). Scores for perceived susceptibility and perceived severity were broken down into three groups: low (0‐3), moderate (4‐6) and high (7‐10).

## 3 RESULTS

### 3.1 Study population

Out of the 1720 panel members that opened the survey, 44 (3%) did not meet the inclusion criteria, 176 (10%) did not consent to participate, and 56 (3%) provided consent but did not complete the survey, giving us a final sample of 1444 adults and a completion rate of 84%.

The sociodemographic and clinical characteristics of the study population are presented in Table 2, and national estimates from the Australian Bureau of Statistics are included for comparison. Approximately 36% of participants were considered high risk for influenza either due to age or the presence of a high‐risk chronic health condition; 23% were aged 65 and over and 21% had at least one high‐risk health condition. More than half of participants (52%) reported that they received an influenza vaccine in 2019. We estimated vaccine coverage to be 44% for adults under 45, 46% for adults aged 45 to 64 and 77% for adults aged 65 and over.

**Table 2:** Sociodemographic and clinical characteristics of study participants (n = 1444)

	Frequency (%)	2019 Australian population^a^ (N = 25 464 116)
Female	775 (54)	50%
Age		
<45	645 (45)	47%
45‐64	462 (32)	32%
65+	337 (23)	21%
State/territory of residence		
New South Wales	436 (30)	32%
Victoria	378 (26)	26%
Queensland	309 (21)	20%
South Australia	124 (9)	7%
Western Australia	141 (10)	10%
Tasmania	35 (2)	2%
Australian Capital Territory	18 (1)	2%
Northern Territory	3 (<1)	1%
Education		
Year 12 or less	460 (32)	41%
TAFE/technical diploma	484 (34)	27%
Tertiary degree	489 (34)	28%
Income		
<$37 000	350 (26)	‐
$37 000+	990 (74)	‐
Born in Australia	1088 (75)	70%
Aboriginal/Torres Strait Islander	53 (4)	3%
Private health insurance	754 (53)	53%^b^
Visited GP in previous 12 mo	1245 (86)	83%
Frequency of pharmacy visits		
3 visits per year or less	522 (36)	‐
More than 3 visits per year	922 (64)	‐
Current smoker	358 (25)	14%
At least 1 high‐risk comorbidity^c^	302 (21)	‐
Influenza vaccination in 2019^c^	747 (53)	‐

^a^ Source: Australian Bureau of Statistics. ^48^

^b^ Source: Australian Institute of Health and Welfare.^49^

^c^ High‐risk comorbidities were self‐reported and include cardiovascular disease, diabetes, history of stroke, immunocompromising conditions, alcohol dependence, chronic kidney or liver disease, and brain or spinal cord conditions.

^d^ Self‐reported.

### 3.2 Self‐reported influenza vaccination in 2019 by sociodemographic and clinical factors

We estimated the prevalence ratios of self‐reported influenza vaccination in 2019 by various sociodemographic and clinical predictors, as illustrated in Table 3.

**Table 3:** Sociodemographic and clinical predictors of self‐reported influenza vaccination in 2019

	Vaccinated In 2019			
	Yes (n = 747)	No (n = 672)	PR^a^ (95% CI)	aPR^b^ (95% CI)
Gender				
Male/other	381 (58%)	276 (42%)	—	—
Female	365 (48%)	395 (52%)	0.83 (0.75, 0.91)^**^	0.94 (0.85, 1.04)
Age				
<45	276 (44%)	351 (56%)	0.95 (0.83,1.09)	0.97 (0.85, 1.10)
45‐64	211 (46%)	245 (54%)	—	—
65+	260 (77%)	76 (23%)	1.67 (1.49, 1.88)^**^	1.49 (1.33, 1.67)^**^
Education				
Year 12 or less	230 (51%)	220 (49%)	—	—
TAFE/technical diploma	242 (51%)	237 (49%)	0.99 (0.87, 1.12)	0.98 (0.88, 1.11)
Tertiary degree	270 (56%)	210 (44%)	1.10 (0.98, 1.24)	1.13 (1.00, 1.26)^*^
Income				
<$37 000	174 (51%)	167 (49%)	—	—
$37 000+	526 (54%)	450 (46%)	1.05 (0.94, 1.19)	1.11 (0.99, 1.25)
Born in Australia				
No	167 (48%)	180 (52%)	—	—
Yes	580 (54%)	492 (46%)	1.12 (0.99, 1.27)	1.13 (1.01, 1.27)^*^
Aboriginal/Torres strait islander				
No	642 (48%)	21 (40%)	—	**—**
Yes	709 (52%)	32 (60%)	1.15 (0.92,1.44)	**1.33 (1.05, 1.68)**
Private health insurance				
No	310 (47%)	356 (53%)	—	—
Yes	430 (58%)	310 (42%)	1.24 (1.13, 1.38)^**^	1.34 (1.18, 1.51)^**^
Visited GP in previous 12 mo				
No	65 (34%)	125 (66%)	**—**	**—**
Yes	682 (56%)	547 (44%)	1.62 (1.32, 1.99)^**^	1.40 (1.14, 1.71)^**^
Frequency of pharmacy visits				
3 Visits per year or less	201 (40%)	308 (60%)	**—**	**—**
More than 3 visits per year	546 (60%)	364 (40%)	1.52 (1.35, 1.71)^**^	1.31 (1.16, 1.19)^**^
Current Smoker				
No	608 (57%)	461 (43%)	**—**	**—**
Yes	139 (40%)	211 (60%)	0.70 (0.61, 0.80)^**^	0.77 (0.67, 0.88)^**^
At least 1 high‐risk comorbidity^c^				
No	528 (47%)	590 (53%)	—	—
Yes	219 (73%)	82 (27%)	1.54 (1.40, 1.69)^**^	1.63 (1.42, 1.88)^**^

^*^p<0.0 ^**^p<0.01

^a^ Prevalence ratio estimated using Poisson regression with robust error estimation.

^b^ Adjusted prevalence ratio; adjusted for age, having private health insurance, GP visit in the previous 12 mo, smoking status, being married and having a high‐risk chronic comorbidity.

^c^ High‐risk comorbidities are self‐reported and include cardiovascular disease, diabetes, history of stroke, immunocompromising conditions, alcohol dependence, chronic kidney or liver disease, and brain or spinal cord conditions.

The prevalence of self‐reported influenza vaccination was significantly higher among those aged 65 and over compared to those aged 45‐64, after adjusting for significant predictors of vaccination (adjusted prevalence ratio (aPR) = 1.49; 95% CI = 1.33‐1.67). There was no significant difference between those aged 45‐64 and those under 45 (aPR = 0.97; 95% CI = 0.85‐1.10). Compared to those with Year 12 completion or less, those with a tertiary degree had a greater prevalence of self‐reported vaccination (aPR = 1.13; 95% CI = 1.00‐1.26), but we did not observe any significant difference between those with a technical degree and those with Year 12 completion. Participants that were born in Australia had a greater prevalence of self‐reported vaccination compared with those born elsewhere (aPR = 1.13; 95% CI = 1.01‐1.27). The prevalence of self‐reported vaccination was greater among those with private health insurance compared with those without (aPR = 1.34; 95% CI = 1.18‐1.51) and was also significantly higher among those that visited a GP in the previous 12 months compared with those that did not (aPR = 1.40; 95% CI = 1.14‐1.71). Participants that reported visiting a pharmacy more than 3 times per year had significantly greater prevalence of self‐reported vaccination compared with those that had three or fewer visits to a pharmacy (aPR = 1.31; 95% CI = 1.16‐1.19). The prevalence of self‐reported vaccination was significantly lower among current smokers compared with those that do not smoke currently (aPR = 0.77; 95% CI = 0.67‐0.88). Other than being over age 65, the strongest single predictor of self‐reported vaccination was having at least one high‐risk chronic health condition (aPR = 1.63; 95% CI = 1.42‐1.88).

### 3.3 Health belief model dimensions

The frequencies of agreement with the HBM dimensions are described in Table 4 for the full sample and by self‐reported influenza vaccination status in 2019.

**Table 4:** Frequencies of agreement with health belief model dimensions by self‐reported influenza vaccination status in 2019

HBM Dimensions	Total (n = 1444)	Vaccinated in 2019			
		Yes (n = 747)	No (n = 672)	PR^a^ (95% CI)	aPR^b^ (95% CI)
Perceived susceptibility^c^					
Low (0‐3)	620 (43%)	280 (37%)	330 (49%)	1.10 (0.96,1.27)	0.99 (0.87, 1.14)
Moderate (4‐6)	442 (31%)	179 (24%)	251 (37%)	Ref	Ref
High (7‐10)	382 (26%)	288 (39%)	91 (14%)	**1.83 (1.61, 2.07)** **	**1.69 (1.50, 1.91)** **
Perceived severity^d^					
Low (0‐3)	453 (31%)	213 (29%)	232 (35%)	0.98 (0.87, 1.12)	1.00 (0.89, 1.13)
Moderate (4‐6)	631 (44%)	300 (40%)	317 (47%)	Ref	Ref
High (7‐10)	360 (25%)	234 (31%)	123 (18%)	**1.35 (1.21, 1.51)** **	**1.29 (1.16, 1.44)**
Perceived benefits					
The flu jab is effective at preventing the flu and keeping me well	1115 (77%)	696 (93%)	404 (60%)	**3.96 (3.06, 5.11)** **	**3.71 (2.87, 4.80)** **
If I get a flu jab, it will protect my loved ones from the flu	887 (61%)	522 (70%)	350 (52%)	**1.46 (1.30, 1.63)** **	**1.53 (1.38, 1.70)** **
Perceived barriers					
Psychological barriers					
The flu jab can give you the flu	535 (36%)	170 (23%)	343 (51%)	**0.52 (0.46, 0.59)** **	**0.59 (0.52, 0.67)** **
The flu jab can make you feel sick afterwards	909 (63%)	377 (50%)	513 (76%)	**0.61 (0.55, 0.67)** **	**0.68 (0.62, 0.74)** **
I don't trust vaccines	196 (14%)	40 (5%)	150 (22%)	**0.37 (0.28, 0.48)** **	**0.42 (0.32, 0.55)** **
Myself or someone I know has had a bad experience with a vaccine	391 (27%)	139 (19%)	241 (36%)	**0.63 (0.54, 0.72)** **	**0.67 (0.58, 0.77)** **
I prefer to develop immunity naturally	526 (36%)	118 (16%)	394 (59%)	**0.33 (0.28, 0.39)** **	**0.38 (0.32, 0.45)** **
I am afraid of needles	439 (30%)	181 (24%)	247 (37%)	**0.74 (0.65, 0.84)** **	**0.84 (0.75, 0.95)** **
Physical/structural barriers					
I have difficulties getting an appointment with my doctor to get vaccinated	173 (12%)	74 (10%)	95 (14%)	**0.81 (0.68, 0.97)** *	0.97 (0.81, 1.16)
Getting the flu jab is expensive	280 (19%)	80 (11%)	190 (28%)	**0.51 (0.42, 0.62)** **	**0.63 (0.52, 0.76)** **
I do not have time to get vaccinated	226 (16%)	76 (10%)	144 (21%)	**0.62 (0.51, 0.75)** **	**0.73 (0.60, 0.89)** **
I have mobility issues that make it difficult to get to my GP	115 (8%)	54 (7%)	56 (8%)	0.93 (0.76, 1.13)	1.00 (0.82, 1.21)
Cues to action					
My doctor recommended that I get the flu jab	840 (58%)	610 (82%)	225 (33%)	**3.11 (2.67, 3.63)** **	**2.70 (2.31, 3.16)** **
I have seen advertisements for the flu jab in pharmacies/chemists	1141 (79%)	651 (87%)	476 (71%)	**1.76 (1.48, 2.09)** **	**1.52 (1.29, 1.80)** **

^a^ Prevalence ratio (PR) estimated using Poisson regression with robust error estimation.

^b^ Adjusted prevalence ratio; adjusted for age, having private health insurance, GP visit in the previous 12 mo, smoking status, frequency of pharmacy visits and having a high‐risk chronic comorbidity.

^c^ Participants answered on a scale from 0 to 10, “how likely do you think you are to get the flu next year?”.

^d^ Participants answered on a scale from 0 to 10, “how severe do you think the flu would be if you got it?”.

* *p*‐value < .05.

** *p*‐value < .01.

For perceived susceptibility, 43% of participants perceived their susceptibility as low, 31% perceived their susceptibility as moderate, and 26% perceived their susceptibility as high. Having low perceived susceptibility to disease was not associated with self‐reported vaccination (aPR = 0.99; 95% CI = 0.87‐1.14) compared with moderate perceived susceptibility, but having high perceived susceptibility was (aPR = 1.69; 95% CI = 1.50‐1.91). For perceived severity, 31% perceived the severity of influenza as low, 44% perceived it as moderate, and 25% perceived it as high. Self‐reported vaccination did not differ between those with low and moderate perceived severity of influenza, but individuals with high perceived severity were significantly more likely to be vaccinated than those with moderate perceived severity (aPR = 1.29; 95% CI = 1.16‐1.44).

In terms of perceived benefits, 77% agreed with “the flu jab is effective at preventing the flu and keeping me well,” which was associated with increased rates of self‐reported vaccination (aPR = 3.71; 95% CI = 2.87‐4.80). 61% agreed with “if I get a flu jab, it will protect my loved ones from the flu,” and they were significantly more likely to self‐report vaccination than those who disagreed (aPR = 1.53; 95% CI = 1.38‐1.70).

For perceived barriers, we included six items that we characterised as “psychological barriers” and four items that we classified as “physical/structural barriers.” There were high rates of agreement with items classified as “psychological barriers,” and all were associated with decreased rates of self‐reported influenza vaccination. While 36% of participants agreed that “the flu jab can give you the flu” (aPR = 0.59; 95% CI = 0.52‐0.67), 63% agreed that “the flu jab can make you feel sick afterwards” (aPR = 0.67; 95% CI = 1.23‐0.74). Fourteen per cent of participants agreed with “I don't trust vaccines” (aPR = 0.42; 95% CI = 0.32‐0.55), and 36% agreed with “I prefer to develop immunity naturally” (aPR = 0.38; 95% CI = 0.32‐0.45). Twenty‐seven per cent of participants agreed with “Myself or someone I know has had a bad experience with a vaccine” (aPR = 0.67; 95% CI = 0.58‐0.77). Thirty per cent of participants agreed with “I am afraid of needles” (aPR = 0.84; 0.75‐0.95). There was less agreement with items classified as “physical/structural barriers.” Only 12% of participants reported that they had difficulties getting an appointment with their doctor to get vaccinated, and only 8% agreed with “I have mobility issues that make it difficult to get to my GP,” and neither was associated with self‐reported influenza vaccination. Nineteen per cent of participants agreed with “getting the flu jab is expensive,” which was associated with decreased self‐reported uptake of the influenza vaccine (aPR = 0.63; 95% CI = 0.52‐0.76). Sixteen per cent of participants agreed with “I do not have time to get vaccinated,” and those that agreed had significantly lower prevalence of self‐reported influenza vaccination (aPR = 0.73; 95% CI = 0.60‐0.89).

Although 86% of participants reported that they had visited a GP in the previous 12 months, only 58% of participants reported that their doctor recommended the influenza vaccine to them, which was strongly associated with increased self‐reported vaccination (aPR = 2.70; 95% CI = 2.31‐3.16). However, 79% recalled having seen advertisements for the influenza vaccine in pharmacies, which was also associated with increased rates of self‐reported vaccination (aPR = 1.52; 95% CI = 1.29‐1.80).

### 3.4 Multivariable Poisson regression

The results from the five HBM constructs are summarised in Table 5. In the multivariable model, high perceived susceptibility to influenza was still significantly predictive of self‐reported influenza vaccination (PR = 1.39; 95% CI = 1.23‐1.56) compared with moderate perceived susceptibility, but high perceived severity of influenza was not (PR = 1.01; 95% CI = 0.91‐1.11). Increasing perceived benefits of vaccination was associated with increased self‐reported vaccination (PR = 1.25; 95% CI = 1.16‐1.35). The likelihood of self‐reported influenza vaccination decreased significantly with increasing levels of perceived psychological barriers (PR = 0.84; 95% CI = 0.82‐0.87), but not perceived physical/structural barriers (PR = 0.97; 95% CI = 0.90, 1.04). Both items related to cues to action remained significantly predictive of self‐reported vaccination (PR = 2.22; 95% CI = 1.90‐2.58 and PR = 1.23; 95% CI = 1.07‐1.42).

**Table 5:** Multivariable Poisson regression indicating associations between health belief model dimensions and self‐reported influenza vaccination in 2019

HBM Dimensions	PR^a^ (95% CI)
Perceived susceptibility	
Low	1.09 (0.96, 1.23)
Moderate	*Ref*
High	1.39 (1.23, 1.56)**
Perceived severity	
Low	1.00 (0.90, 1.10)
Moderate	*Ref*
High	1.01 (0.91, 1.11)
Perceived benefits	1.25 (1.16, 1.35)**
Perceived barriers	
Psychological barriers	0.84 (0.82, 0.87)**
Physical/structural barriers	0.97 (0.90, 1.04)
Cues to action	
My doctor recommended that I get the flu jab	2.22 (1.90, 2.58)**
I saw advertisements for the flu jab in pharmacies/chemists	1.23 (1.07, 1.42)**

^a^ Prevalence ratio (PR) estimated using Poisson regression with robust error estimation.

* *p*‐value < .05

** *p*‐value < .01

## 4 DISCUSSION

We found that influenza vaccination coverage among Australian adults was higher than in previous years but remains sub‐optimal. The most recent nation‐wide survey in 2014 found that approximately 40% of adults received the influenza vaccine that year,^6^ but in our study 44% of adults under 45, 46% of adults aged 45 to 64 and 77% of adults aged 65 and over reported that they received the influenza vaccine in 2019. While this is encouraging, certain adults were less likely to report being vaccinated for influenza, such as adults under age 65, smokers and those born outside Australia. Furthermore, we identified a number of barriers that may be preventing more widespread uptake of influenza vaccines.

A systematic review of influenza vaccine coverage in Australia conducted in 2015 found that the most important predictor of influenza vaccination was being aged 65 and over,^11^ which has also been observed in other countries.^3^ In our study, the prevalence of self‐reported vaccination was nearly 50% higher among those over 65 compared with those aged 45‐64, even after adjusting for health status and healthcare utilisation factors. In Australia, universal funding for influenza vaccination of adults aged 65 and over was introduced in 1999 and resulted in a significant increase in vaccine coverage for this group.^11^ Given the success and cost‐effectiveness of this programme,^26^ some have argued that universal funding should be expanded to include adults aged 50 to 64. While previous studies found mixed results in terms of cost‐effectiveness,^27^, ^28^ they did not include increased risk of myocardial infarction or other secondary outcomes of influenza infection.^29^, ^30^ We found that vaccination coverage was low in this age group; less than half of adults aged 45 to 64 were vaccinated for influenza in 2019. New economic models should be developed to determine whether universal vaccination funding should be implemented for this group.

In Australia, influenza vaccination is also funded for anyone with a high‐risk chronic health condition. While people that smoke tobacco are considered high risk, they are not included in the funding scheme.^31^ We found that participants that were daily smokers were significantly less likely to report being vaccinated for influenza in 2019. Similarly, a survey of Australian adults over age 45 with chronic conditions also found that smokers were significantly less likely to get vaccinated for influenza.^32^ However, adults that smoke tobacco are more likely to develop severe influenza requiring hospitalisation compared with non‐smokers.^33^, ^34^ Therefore, targeted interventions are needed in this group to promote uptake, such as including smokers in the National Immunisation Program.

In our study, adults with at least one chronic health condition were significantly more likely to report influenza vaccination in 2019 compared with those without chronic conditions. While this may be due in part to universal influenza vaccination funding for this group, there are several other explanations as well. Adults with chronic conditions are more likely to have high perceived risk of disease, receive a recommendation from their healthcare providers and visit pharmacies more regularly, which were predictors of influenza vaccination in this study. Although 73% of adults with chronic conditions in our study reported influenza vaccination in 2019, modelling suggests that at least 90% of high‐risk individuals should be vaccinated for influenza annually to achieve herd immunity.^35^ Further research is needed to determine how best to increase uptake in younger adults with chronic health conditions. Alternatively, improving influenza vaccine uptake among children may provide indirect protection for high‐risk adults.^36^

We used the HBM to better understand vaccine decision‐making among Australian adults. According to this model, the likelihood of an individual engaging in a health‐promoting behaviour, such as vaccination, is determined in part by their perceptions of how severe a health threat is and how likely they are to experience that threat.^21^ Both high perceived susceptibility and high perceived severity were significant predictors of self‐reported influenza vaccination in our study, even after adjusting for age and health status. However, having low perceived susceptibility and severity were not predictive of vaccination compared with moderate perceived susceptibility and severity. Perceived susceptibility to influenza is typically low. In 2009, more than half of Australians perceived their risk of contracting pandemic influenza as low or very low, and these adults were less likely to report willingness to accept the vaccine compared with those that believed they were at high risk.^10^ We found that perceived severity was no longer significant after adjusting for other HBM dimensions; however, perceived severity is typically not as strongly associated with preventative health behaviours.^37^

According to the HBM, engagement in a health‐promoting behaviour is also predicted by an individual's perceived benefits of the intervention, which includes their perceived effectiveness of it.^21^ In our study, one of the strongest predictors of self‐reported influenza vaccination was the belief that vaccination was effective at preventing influenza. The belief that getting the influenza vaccine would help protect one's loved ones was also significantly associated with self‐reported vaccination. These findings were consistent with the results of a 2007 survey which found that the odds of influenza vaccination among Australian adults aged 40‐64 were nearly five times greater among those that believed the vaccine was effective.^12^ This is troubling when considering that nearly one in four participants in our study did not believe that the vaccine was effective, and nearly 40% of participants did not believe that getting vaccinated for influenza would protect their loved ones from getting sick. The effectiveness of seasonal influenza vaccines is moderate and highly variable year to year,^38^ which may impact public confidence in the vaccine.

In addition, we identified several perceived barriers that may be contributing to low vaccine coverage among Australian adults. Worryingly, nearly 40% of people believe that the influenza vaccine can give you influenza and are significantly less likely to get vaccinated, despite the fact that there are no documented instances of influenza vaccine virus reverting to wild‐type virus and causing disease.^39^ However, this has proven to be a difficult vaccine myth to dispel; research suggests that correcting this myth does not increase willingness to receive the vaccine and may in fact have the opposite effect.^40^ In general, messaging that advocates for vaccination too strongly can actually bolster vaccine hesitancy rather than alleviate it.^41^ Fear of side effects in general appears to be a significant barrier, given that two‐thirds of adults agree that the vaccine can make you feel sick afterwards, and are significantly less likely to report taking the vaccine. As mentioned above, perceived susceptibility to influenza is generally low, and this is likely affecting how consumers are weighing the risks and benefits of the influenza vaccine. For an individual that perceives their risk of influenza as non‐existent or very low, it may be outweighed by the risk of even mild adverse events following vaccination. Thus, it may not just be a question of dispelling harmful vaccine myths, but also conveying the benefits of vaccination.

By contrast, physical and structural barriers of vaccination were not commonly reported. For example, 12% of participants reported having difficulties getting an appointment and only 16% felt they did not have time. Furthermore, when we adjusted for psychological predictors, physical barriers did not significantly predict vaccination. Thus, unlike other routine childhood vaccines,^42^ low influenza vaccine coverage among Australian adults appears to be caused primarily by individual beliefs and attitudes, rather than issues with access.

Given the complexity of the psychological determinants of influenza vaccination, the most effective solution may be individually tailored health education.^41^ This approach has been effective in the context of parental vaccine hesitancy. For example, MomTalkShots is an application that delivers educational videos that are algorithmically tailored to an individual's demographics and vaccine attitudes, beliefs and intentions.^43^ Presently, the responsibility of delivering vaccine messaging and education typically falls on GPs. While there may be some recall bias involved, we found that participants were significantly more likely to report vaccination if their GP specifically recommending the vaccine. However, physicians may not be adequately prepared to deal with the complexities of vaccine hesitancy, they may lack the time to determine an individual's specific beliefs about vaccination,^44^ and they may have their own hesitancy towards vaccination.^45^ Tools should be developed to help vaccine providers, such as GP’s and pharmacists, to tailor their recommendations on influenza vaccination to a patient's individual attitudes, beliefs and intentions. A similar platform to MomTalkShots could be developed for adult vaccination, which could be utilised by GP’s when organizing annual influenza vaccine clinics. This, coupled with SMS reminders, may encourage patients to receive annual vaccination.^46^ Uptake may also be improved by an “active choice” model where vaccine providers are automatically asked whether they want to order a vaccine for their patient when filling out their electronic medical record.^44^

This study was not without limitations. There were several potential sources of bias. Since panel members were provided the survey link and given the option to open it, those that chose to participate in the survey may differ from those that chose not to participate. Furthermore, panel members may not be truly representative of the Australian population,^47^ and thus, our estimates of vaccine coverage need to be interpreted with caution. Finally, self‐reported variables, such as vaccination status and healthcare provider recommendation, are susceptible to response or recall bias.

## CONFLICT OF INTEREST

Dr C Raina MacIntyre is receiving funding from Sanofi for influenza burden of disease research. Dr Daniel Salmon has had consulting and/or grant support from Merck, Walgreens and Pfizer. Mallory J Trent has no competing interests to declare.

## AUTHOR CONTRIBUTIONS

**Mallory Trent:** Conceptualization (equal); Data curation (equal); Formal analysis (lead); Methodology (equal); Project administration (equal); Writing‐original draft (lead). **Daniel A. Salmon:** Conceptualisation (equal); methodology (equal); formal analysis (supporting); supervision (equal) and writing‐review & editing (equal). **C. Raina MacIntyre:** Conceptualization (equal); Funding acquisition (lead); Methodology (equal); Supervision (lead); Writing‐review & editing (equal).

### PEER REVIEW

The peer review history for this article is available at https://publons.com/publon/10.1111/irv.12843.

## Supporting information

Supplementary Material

## Data Availability

Research data are not shared.
