# Supplementary material for: Using the health belief model to identify barriers to seasonal influenza vaccination among Australian adults in 2019
Source: Influenza Other Respir Viruses. 2021 Feb 15;15(5):678–87. doi: 10.1111/irv.12843 (PMC8404057; doi:10.1111/irv.12843)
Supplement: Supplementary file 1 — Supplementary Material [file IRV-15-678-s001.docx]

Supplementary Information – Survey Questions

## Healthcare Utilisation

*The following questions will ask you about your healthcare providers. Please select the most appropriate response to each question.*

1. In the past 12 months, which of the following have you visited for treatment or advice about your health? (Please select all that apply)
   1. General practitioner (GP) / Family Doctor
   2. Specialist doctor
   3. Medical center
   4. Chemist/Pharmacy
   5. Workplace
   6. Hospital
   7. Aboriginal Medical Service (AMS)
   8. Other [specify]
   9. None
2. Do you have a regular GP or practice (i.e. that you have visited more than once)?
   1. Yes, I have one regular GP
   2. Yes, I have a regular practice, but I see more than one GP at that practice
   3. No, I do not have a regular GP or medical center
   4. Not sure
3. Do you currently have a valid Australian Medicare card?
   1. Yes
   2. No
   3. Not sure
4. Do you have private health insurance?
   1. Yes
   2. No
   3. Not sure
5. In the past 12 months, about how often did you visit the pharmacy/chemist?
   1. More than once per month
   2. Once per month
   3. Every other month
   4. 2-3 times per year
   5. Once
   6. Never

## Vaccination History

*The following questions will ask you about your vaccination history. Please select the most appropriate response to each question. Please provide additional information where indicated.*

1. Do you get the flu jab (flu vaccine)?
   1. Yes, every year
   2. Yes, some years
   3. No, never
2. Did you get the flu jab in 2019?
   1. Yes
   2. No
   3. Not sure
3. *[If yes]* During which month did you get the flu jab?
   1. *Select month*
4. *[If yes]* Is this the first time you have received a flu jab?
   1. Yes, this was the first time I got the flu jab
   2. No, I had gotten the flu jab before
5. [*If received flu jab in 2019*] Where did you get the jab this year?
   1. GP’s office/surgery
   2. Medical center
   3. Chemist/Pharmacy
   4. Workplace
   5. Hospital
   6. Aboriginal Medical Service (AMS)
   7. Other [specify]
   8. Don’t know —
6. Why did you choose to get vaccinated there? (Please check all that apply)
   1. Convenience
   2. Cost
   3. Trust in the provider
   4. That’s just where I always get the flu jab
   5. Other
7. (Qualitative) Please explain why you chose to get vaccinated there
8. Did you get the flu jab in 2018?
   1. Yes
   2. No
   3. Not sure
9. Have you ever received a vaccine for pneumonia (pneumococcal vaccine)?
   1. Yes
   2. No
   3. Not sure
10. *[If yes*] When did you receive the vaccine for pneumonia (pneumococcal vaccine)?
    - 1. Within the last 12 months
      2. 12 months to 5 years ago
      3. More than 5 years ago
      4. Don't know
11. Have you ever received a vaccine for shingles (Zoster)?
    1. Yes
    2. No
    3. Not sure
12. *[If yes*] When did you receive the vaccine for shingles?
    - 1. Within the last 12 months
      2. 12 months to 5 years ago
      3. More than 5 years ago
      4. Don't know

# Health History

*The following questions will ask you about your current health status. Please select the most appropriate response to each question. Please provide additional information where indicated.*

1. Please indicate if a doctor has ever diagnosed you with any of the following:
   1. High blood pressure
   2. High cholesterol
   3. Heart disease
      1. (drop down to specify i: heart attack (when), Angina but no heart attack, bypass surgery, heart failure, pacemaker, other)
   4. Cancer
      1. specify type
      2. are you currently undergoing treatment?
   5. Chronic lung disease
      1. (drop down to specify if: COPD, emphysema, severe asthma, cystic fibrosis, other)
   6. Diabetes
   7. Weak immune system
      1. (drop down to specify if: HIV, sickle cell disease, removed or dysfunctional spleen, organ transplant, bone marrow transplant)
   8. Stroke
      1. (dropdown to specify if: Stroke (when), TIA’s)
   9. Alcohol dependence
   10. Other
       1. (drop down to specify if: liver disease (specify), kidney disease (specify), brain or spinal cord condition (drop down: multiple sclerosis, seizure, spinal cord injury, other))
2. Are you currently pregnant?
   1. Yes
   2. No
   3. Not sure
3. Have you been pregnant in the last 5 years?
   1. Yes
   2. No
   3. Not sure
4. Do you currently smoke tobacco (cigarettes, cigars, pipe)?
   1. I smoke tobacco daily
   2. I smoke tobacco, but not daily
   3. I do not smoke at all

# Influenza

*The following items describe statements about the flu, pneumonia, and vaccines. Please indicate your agreement or disagreement to each statement by selecting the most appropriate response.*

Perceived Susceptibility

1. On a scale from 1-10, how likely do you think you are to get the flu next year?
   1. 1 (Not likely at all) … somewhat likely…10 (Extremely likely)

Perceived Severity

1. On a scale of 1-10, how severe do you think the flu would be if you got it?
   1. 1 (Not severe at all) … 10 (Extremely severe)

Perceived Benefits

1. The flu jab is effective at preventing the flu and keeping me well
   1. Strongly disagree
   2. Disagree
   3. Agree
   4. strongly agree
2. If I get a flu jab, it will help protect my loved ones from the flu
   1. Strongly disagree
   2. Disagree
   3. Agree
   4. strongly agree

Perceived Barriers

1. The flu jab can give you the flu
   1. Disagree
   2. Agree
2. The flu jab can make you feel sick afterwards
   1. Disagree
   2. Agree
3. I don’t trust vaccines
   1. Strongly disagree
   2. Disagree
   3. Agree
   4. strongly agree
4. Myself or someone I know has had a bad experience with a vaccine
   1. Disagree
   2. Agree
5. I prefer to develop immunity naturally, rather than take vaccines
   1. Strongly disagree
   2. Disagree
   3. Agree
   4. strongly agree
6. I am afraid of needles
   1. Strongly disagree
   2. Disagree
   3. Agree
   4. Strongly agree
7. I have difficulties getting an appointment with my doctor to get vaccinated
   1. Strongly disagree
   2. Disagree
   3. Agree
   4. strongly agree
8. Getting the flu jab is too expensive
   1. Strongly disagree
   2. Disagree
   3. Agree
   4. strongly agree
9. I do not have time to visit my GP to get vaccinated
   1. Disagree
   2. agree
10. I have mobility issues that make it difficult to visit my GP
    1. Disagree
    2. Agree
11. Flu vaccine is safe during pregnancy
    1. Strongly disagree
    2. Disagree
    3. Agree
    4. strongly agree

Cues to action

1. I have a health condition that puts me at increased risk of the flu
   1. Disagree
   2. agree
2. I am eligible for a free influenza vaccine under the government’s National Immunisation Programme
   1. Disagree
   2. agree
3. My doctor has recommended the influenza vaccine to me
   1. Disagree
   2. agree
4. I have seen advertisements for flu jabs in pharmacies/chemists
   1. Disagree
   2. agree
5. I got the flu last year
   1. Disagree
   2. agree

# Pneumococcal Disease

*The following items describe statements about the flu, pneumonia, and vaccines. Please indicate your agreement or disagreement to each statement by selecting the most appropriate response.*

Perceived Susceptibility

1. On a scale from 1 – 10, how likely do you think you are to get pneumonia?
   1. 1 (Not likely at all) ... somewhat likely…. 10 (Extremely likely)

Perceived Severity

1. On a scale from 1 – 10, if you got pneumonia, how severe do you think it would be?
   1. 1 (Not severe at all) ... Somewhat severe…10 (Extremely severe)

Perceived Benefits

1. I believe the pneumonia vaccine is effective at preventing pneumonia
   1. Strongly disagree
   2. Disagree
   3. Agree
   4. strongly agree

Perceived Barriers

1. I have never heard of the pneumonia vaccine before
   1. Disagree
   2. agree

Cues to action

1. I have a health condition that puts me at higher risk of developing pneumonia
   1. Disagree
   2. agree
2. I am eligible for a free pneumonia vaccine under the government’s national immunisation program
   1. Disagree
   2. agree
3. My doctor has recommended the pneumonia vaccine to me
   1. Disagree
   2. agree
4. I have had pneumonia in the past
   1. Disagree
   2. agree

# Sociodemographic information

*The following questions will ask you about you and your background. Please select the most appropriate response to each question.*

1. What is your age?
   1. 18-24
   2. 25-34
   3. 35-44
   4. 45-54
   5. 55-64
   6. 65-74
   7. 75-84
   8. 85 and over
2. What is your gender?
   1. Female
3. Male
4. Non-binary/Third gender
5. Other (please describe)
6. Prefer not to say
7. What state/territory do you reside in?
8. New South Wales
9. Victoria
10. Queensland
11. South Australia
12. Western Australia
13. Tasmania
14. Northern Territory
15. Australian Capital Territory
16. What is your highest level of education completed? (Note: If you’re currently in school, please indicate the highest degree you have *received*)
17. Completed Year 10 or less
18. Completed Year 12 or equivalent
19. TAFE or vocational/technical diploma
20. Bachelor’s degree (Honours included)
21. Master’s degree
22. Doctorate
23. Which of the following best describes your current work status?
24. Employed, Full time
25. Employed, Part time
26. Employed, Casual
27. Self-employed/Freelance
28. Homemaker
29. Military/ Armed Forces
30. Unemployed, looking for work
31. Unemployed, not looking for work
32. Retired
33. Studying
34. Not able to work due to a disability
35. Other (please describe)
36. Do you work in a profession that involves caring for other people (e.g. AHPRA registered health worker, aged care worker, childcare worker, disability or long-term care worker)?
    1. Yes
    2. No
37. What is your approximate annual household income before taxes from all sources (including pension, superannuation, etc)?
38. Under $18,200
39. $18,201 to $37,000
40. $37,001 – $90,000
41. $90,001 – $180,000
42. Over $180,000
43. What is your country of birth?
44. Australia
45. Other (Please specify)
46. Which of the following best describes your current marital status?
47. Single (never married)
48. Married/De facto
49. Divorced
50. Separated
51. Widowed
52. Are you Aboriginal/Torres Strait Islander?
53. Yes, Aboriginal
54. Yes, Torres Strait Islander
55. Yes, both
56. No
57. Not sure
58. Prefer not to say
59. How many people live in your household?

*a. Enter #*

1. How many members of your household are under age 5?
2. *Enter #*
